# Trichlorido(dimethyl sulfoxide-κ*O*)(di-2-pyridyl­amine-κ^2^ *N*,*N*′)indium(III)

**DOI:** 10.1107/S1600536812038147

**Published:** 2012-09-12

**Authors:** Sadif A. Shirvan, Sara Haydari Dezfuli, Elyas Golabi

**Affiliations:** a Department of Chemistry, Islamic Azad University, Omidieh Branch, Omidieh, Iran; b Department of Petroleum Engineering, Omidieh Branch, Islamic Azad University, Omidieh, Iran

## Abstract

In the title compound, [InCl_3_(C_10_H_9_N_3_)(C_2_H_6_OS)], the In^III^ atom is six-coordinated in a distorted octa­hedral geometry by two N atoms from a chelating di-2-pyridyl­amine ligand, one O atom from a dimethyl sulfoxide ligand and three Cl atoms. Inter­molecular C—H⋯Cl hydrogen bonds and π–π contacts between the pyridine rings [centroid–centroid distance = 3.510 (3) Å] are present in the crystal.

## Related literature

For related structures, see: Abedi *et al.* (2011 ▶, 2012*a* ▶,*b* ▶); Ahmadi *et al.* (2008 ▶); Clemente (2005 ▶); Dong *et al.* (1987 ▶); Ilyuhin & Malyarik (1994 ▶); Kalateh, Ahmadi *et al.* (2008 ▶); Kalateh, Norouzi *et al.* (2008 ▶); Malecki *et al.* (2011 ▶); Malyarick *et al.* (1992 ▶); Shi & Jiang (2006 ▶); Shirvan & Haydari Dezfuli (2012 ▶); Yoshikawa *et al.* (2004 ▶); Yousefi *et al.* (2009 ▶).
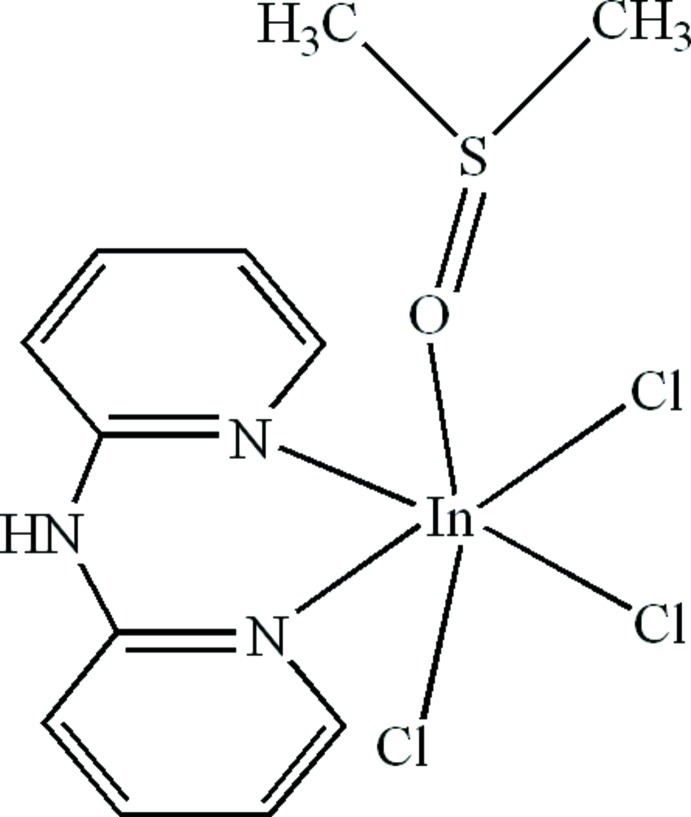


## Experimental

### 

#### Crystal data

[InCl_3_(C_10_H_9_N_3_)(C_2_H_6_OS)]
*M* *_r_* = 470.51
Monoclinic, 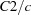

*a* = 29.283 (2) Å
*b* = 7.7642 (7) Å
*c* = 15.9459 (12) Å
β = 104.891 (6)°
*V* = 3503.7 (5) Å^3^
*Z* = 8
Mo *K*α radiation
μ = 1.93 mm^−1^
*T* = 298 K
0.20 × 0.18 × 0.15 mm

#### Data collection

Bruker APEXII CCD diffractometer
Absorption correction: multi-scan (*SADABS*; Bruker, 2001 ▶) *T*_min_ = 0.702, *T*_max_ = 0.796
14020 measured reflections
3448 independent reflections
2503 reflections with *I* > 2σ(*I*)
*R*_int_ = 0.075

#### Refinement

*R*[*F* ^2^ > 2σ(*F* ^2^)] = 0.042
*wR*(*F* ^2^) = 0.080
*S* = 0.99
3448 reflections
192 parameters
H-atom parameters constrained
Δρ_max_ = 0.72 e Å^−3^
Δρ_min_ = −0.61 e Å^−3^

### 

Data collection: *APEX2* (Bruker, 2007 ▶); cell refinement: *SAINT* (Bruker, 2007 ▶); data reduction: *SAINT*; program(s) used to solve structure: *SHELXS97* (Sheldrick, 2008) ▶; program(s) used to refine structure: *SHELXL97* (Sheldrick, 2008) ▶; molecular graphics: *ORTEP-3* (Farrugia, 1997 ▶); software used to prepare material for publication: *SHELXTL* (Sheldrick, 2008) ▶.

## Supplementary Material

Crystal structure: contains datablock(s) I, global. DOI: 10.1107/S1600536812038147/hy2584sup1.cif

Structure factors: contains datablock(s) I. DOI: 10.1107/S1600536812038147/hy2584Isup2.hkl

Additional supplementary materials:  crystallographic information; 3D view; checkCIF report

## Figures and Tables

**Table 1:** Hydrogen-bond geometry (Å, °)

*D*—H⋯*A*	*D*—H	H⋯*A*	*D*⋯*A*	*D*—H⋯*A*
C11—H11*C*⋯Cl2^i^	0.96	2.74	3.499 (8)	137

Symmetry code: (i) 
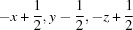
.

**Fig. 1.**
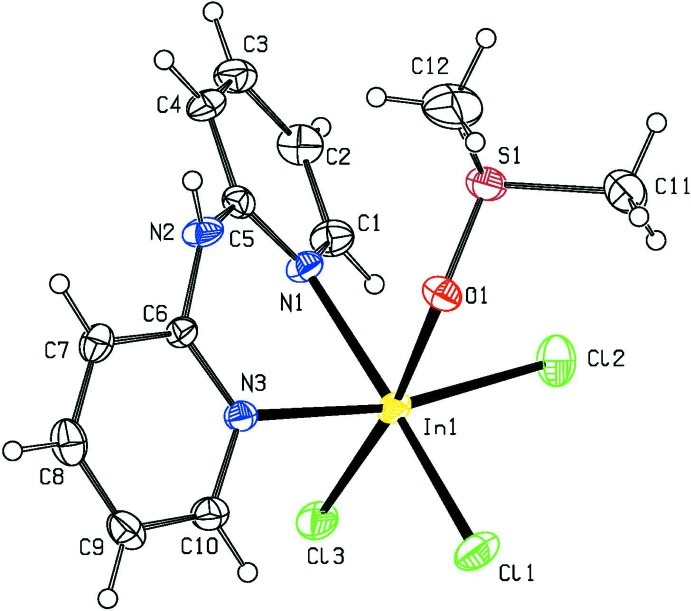
The molecular structure of the title compound. Displacement ellipsoids are drawn at the 30% probability level.

**Fig. 2.**
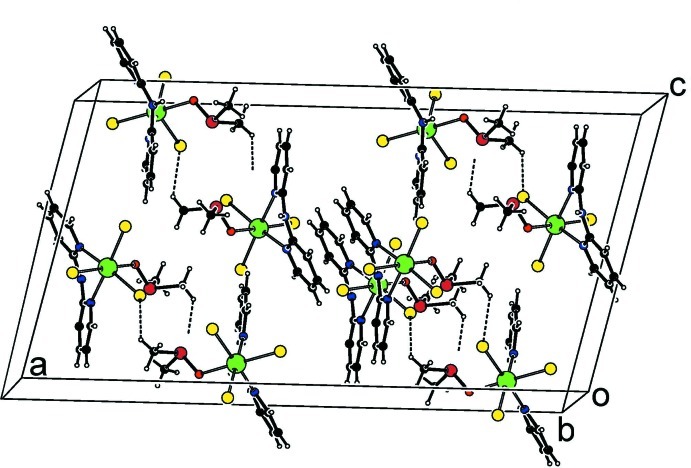
A packing diagram of the title compound. Hydrogen bonds are shown as dashed lines.

## supplementary crystallographic information

### Comment

Recently, we reported the synthesis and crystal structure of [In(4,4'-dmbipy)Cl_3_(MeOH)].MeOH, (II) (Shirvan & Haydari Dezfuli, 2012) (4,4'-dmbipy = 4,4'-dimethyl-2,2'-bipyridine). Several In^III^ complexes with a formula [In(*L*_1_)Cl_3_(*L*_2_)] (*L*_1_ = an N,N'-chelating ligand, *L*_2_ = DMSO, H_2_O, MeOH or EtOH), such as [In(bipy)Cl_3_(H_2_O)], (III), [In(bipy)Cl_3_(EtOH)], (IV), [In(bipy)Cl_3_(H_2_O)].H_2_O, (V) (Malyarick *et al.*, 1992), [In(phen)Cl_3_(DMSO)], (VI) (Dong *et al.*, 1987), [In(phen)Cl_3_(H_2_O)], (VII), [In(phen)Cl_3_(EtOH)].EtOH, (VIII) (Ilyuhin & Malyarik, 1994), [In(4,4'-dmbipy)Cl_3_(DMSO)], (IX) (Ahmadi *et al.*, 2008), [In(5,5'-dmbipy)Cl_3_(MeOH)], (X) (Kalateh, Ahmadi *et al.*, 2008), [In(4,4'-dtbipy)Cl_3_(MeOH)].0.5MeOH, (XI) (Abedi *et al.*, 2012*a*), [In(4bt)Cl_3_(MeOH)], (XII) and [In(4bt)Cl_3_(DMSO)], (XIII) (Abedi *et al.*, 2012*b*) (bipy = 2,2'-bipyridine, phen = 1,10-phenanthroline, DMSO = dimethyl sulfoxide, 4,4'-dmbipy = 4,4'-dimethyl-2,2'-bipyridine, 5,5'-dmbipy = 5,5'-dimethyl-2,2'-bipyridine, 4,4'-dtbipy = 4,4'-di-tert-butyl-2,2'-bipyridine, 4bt = 4,4'-bithiazole), have been synthesized and characterized by single-crystal X-ray diffraction methods. Di-2-pyridylamine (DPA) is a good bidentate ligand, and numerous complexes with DPA have been prepared, such as that of [Hg(DPA)Br_2_], (XIV) (Kalateh, Norouzi *et al.*, 2008), [Hg(DPA)Cl_2_], (XV) (Yousefi *et al.*, 2009), [Pt(DPA)Cl_4_].DMF, (XVI) (Abedi *et al.*, 2011), [Ir(DPA)_2_Cl_2_](PF_6_), (XVII) (Yoshikawa *et al.*, 2004), [Cu(DPA)_2_](BF_4_)_2_, (XVIII) (Clemente, 2005), [Mn(DPA)_2_(NCS)_2_].0.5H_2_O, (XIX) (Malecki *et al.*, 2011) and [Au(DPA)Cl_2_]Cl, (XX) (Shi & Jiang, 2006). We report herein the synthesis and crystal structure of the title compound, (I).

In the title compound (Fig. 1), the In^III^ atom is six-coordinated in a distorted octahedral geometry by two N atoms from a chelating DPA ligand, one O atom from a dimethyl sulfoxide ligand and three Cl atoms. In the crystal, intermolecular C—H···Cl hydrogen bonds (Table 1, Fig. 2) and π–π contacts between the pyridine rings, *Cg*3···*Cg*3^i^ [symmetry code: (i) -*x*, -y, -*z*. *Cg*3 is the centroid of the N3/C6–C10 ring], with a centroid–centroid distance of 3.510 (3) Å, stabilize the structure.

### Experimental

For the preparation of the title compound, a solution of di-2-pyridylamine (0.29 g, 1.65 mmol) in methanol (10 ml) was added to a solution of InCl_3_.4H_2_O (0.48 g, 1.65 mmol) in methanol (10 ml) at room temperature. The suitable crystals for X-ray diffraction analysis were obtained by methanol diffusion into a colorless solution in DMSO after one week (yield: 0.57 g, 73.4%).

### Refinement

All H atoms were positioned geometrically, with C—H = 0.93 (CH), 0.96 (CH_3_) and N—H = 0.86 Å and with *U*_iso_(H) = 1.2*U*_eq_(C, N).

### Crystal data

**Table:**

[InCl_3_(C_10_H_9_N_3_)(C_2_H_6_OS)]	*F*(000) = 1856
*M**_r_* = 470.51	*D*_x_ = 1.784 Mg m^−^^3^
Monoclinic, *C*2/*c*	Mo *K*α radiation, λ = 0.71073 Å
Hall symbol: -C 2yc	Cell parameters from 14020 reflections
*a* = 29.283 (2) Å	θ = 2.6–26.0°
*b* = 7.7642 (7) Å	µ = 1.93 mm^−^^1^
*c* = 15.9459 (12) Å	*T* = 298 K
β = 104.891 (6)°	Block, colorless
*V* = 3503.7 (5) Å^3^	0.20 × 0.18 × 0.15 mm
*Z* = 8	

### Data collection

**Table:**

Bruker APEXII CCD diffractometer	3448 independent reflections
Radiation source: fine-focus sealed tube	2503 reflections with *I* > 2σ(*I*)
Graphite monochromator	*R*_int_ = 0.075
φ and ω scans	θ_max_ = 26.0°, θ_min_ = 2.6°
Absorption correction: multi-scan (*SADABS*; Bruker, 2001)	*h* = −29→36
*T*_min_ = 0.702, *T*_max_ = 0.796	*k* = −9→9
14020 measured reflections	*l* = −19→19

### Refinement

**Table:**

Refinement on *F*^2^	Primary atom site location: structure-invariant direct methods
Least-squares matrix: full	Secondary atom site location: difference Fourier map
*R*[*F*^2^ > 2σ(*F*^2^)] = 0.042	Hydrogen site location: inferred from neighbouring sites
*wR*(*F*^2^) = 0.080	H-atom parameters constrained
*S* = 0.99	*w* = 1/[σ^2^(*F*_o_^2^) + (0.0384*P*)^2^] where *P* = (*F*_o_^2^ + 2*F*_c_^2^)/3
3448 reflections	(Δ/σ)_max_ = 0.007
192 parameters	Δρ_max_ = 0.72 e Å^−^^3^
0 restraints	Δρ_min_ = −0.61 e Å^−^^3^

### Special details

**Table:**

Geometry. All e.s.d.'s (except the e.s.d. in the dihedral angle between two l.s. planes) are estimated using the full covariance matrix. The cell e.s.d.'s are taken into account individually in the estimation of e.s.d.'s in distances, angles and torsion angles; correlations between e.s.d.'s in cell parameters are only used when they are defined by crystal symmetry. An approximate (isotropic) treatment of cell e.s.d.'s is used for estimating e.s.d.'s involving l.s. planes.
Refinement. Refinement of *F*^2^ against ALL reflections. The weighted *R*-factor *wR* and goodness of fit *S* are based on *F*^2^, conventional *R*-factors *R* are based on *F*, with *F* set to zero for negative *F*^2^. The threshold expression of *F*^2^ > σ(*F*^2^) is used only for calculating *R*-factors(gt) *etc*. and is not relevant to the choice of reflections for refinement. *R*-factors based on *F*^2^ are statistically about twice as large as those based on *F*, and *R*- factors based on ALL data will be even larger.

### Fractional atomic coordinates and isotropic or equivalent isotropic displacement parameters (Å^2^)

**Table:**

	*x*	*y*	*z*	*U*_iso_*/*U*_eq_	
C1	0.1269 (2)	0.2012 (6)	0.2648 (3)	0.0416 (13)	
H1	0.1292	0.3198	0.2730	0.050*	
C2	0.1331 (2)	0.0973 (7)	0.3361 (4)	0.0444 (13)	
H2	0.1397	0.1439	0.3916	0.053*	
C3	0.12911 (19)	−0.0815 (7)	0.3234 (4)	0.0422 (13)	
H3	0.1334	−0.1557	0.3705	0.051*	
C4	0.11901 (18)	−0.1440 (6)	0.2417 (3)	0.0363 (12)	
H4	0.1167	−0.2623	0.2325	0.044*	
C5	0.11200 (16)	−0.0330 (6)	0.1712 (3)	0.0301 (10)	
C6	0.07132 (16)	−0.0304 (5)	0.0123 (3)	0.0276 (10)	
C7	0.04239 (18)	−0.1417 (6)	−0.0476 (3)	0.0367 (12)	
H7	0.0439	−0.2600	−0.0381	0.044*	
C8	0.01195 (19)	−0.0754 (7)	−0.1202 (3)	0.0433 (13)	
H8	−0.0075	−0.1478	−0.1605	0.052*	
C9	0.01051 (18)	0.1023 (7)	−0.1329 (3)	0.0415 (13)	
H9	−0.0105	0.1510	−0.1810	0.050*	
C10	0.04049 (17)	0.2029 (6)	−0.0735 (3)	0.0355 (12)	
H10	0.0393	0.3215	−0.0819	0.043*	
C11	0.2689 (2)	0.1898 (9)	0.1207 (5)	0.0667 (19)	
H11A	0.2654	0.3090	0.1337	0.080*	
H11B	0.2735	0.1797	0.0635	0.080*	
H11C	0.2957	0.1425	0.1622	0.080*	
C12	0.2306 (3)	−0.1246 (8)	0.0852 (6)	0.090 (3)	
H12A	0.2358	−0.1087	0.0286	0.108*	
H12B	0.2047	−0.2024	0.0812	0.108*	
H12C	0.2586	−0.1717	0.1236	0.108*	
N1	0.11766 (14)	0.1385 (4)	0.1824 (3)	0.0320 (9)	
N2	0.09923 (15)	−0.1004 (5)	0.0877 (3)	0.0341 (10)	
H2B	0.1104	−0.2010	0.0822	0.041*	
N3	0.07196 (13)	0.1395 (4)	−0.0027 (2)	0.0289 (9)	
O1	0.17759 (11)	0.1461 (4)	0.0516 (2)	0.0371 (8)	
In1	0.124091 (13)	0.32946 (4)	0.07746 (2)	0.02991 (11)	
Cl1	0.12945 (6)	0.49624 (17)	−0.04921 (10)	0.0507 (4)	
Cl2	0.18785 (6)	0.48512 (18)	0.17913 (10)	0.0562 (4)	
Cl3	0.05889 (5)	0.49583 (15)	0.10961 (9)	0.0437 (3)	
S1	0.21736 (5)	0.07577 (18)	0.12573 (10)	0.0435 (3)	

### Atomic displacement parameters (Å^2^)

**Table:**

	*U*^11^	*U*^22^	*U*^33^	*U*^12^	*U*^13^	*U*^23^
C1	0.051 (3)	0.035 (3)	0.038 (3)	0.005 (2)	0.010 (3)	−0.001 (2)
C2	0.054 (4)	0.045 (3)	0.033 (3)	0.005 (3)	0.010 (3)	−0.006 (2)
C3	0.043 (3)	0.047 (3)	0.039 (3)	0.009 (2)	0.013 (3)	0.013 (2)
C4	0.045 (3)	0.021 (2)	0.044 (3)	0.005 (2)	0.012 (2)	0.004 (2)
C5	0.024 (2)	0.030 (2)	0.035 (3)	0.0034 (19)	0.007 (2)	−0.001 (2)
C6	0.029 (3)	0.023 (2)	0.031 (3)	−0.0002 (18)	0.008 (2)	0.0012 (18)
C7	0.042 (3)	0.026 (2)	0.042 (3)	−0.006 (2)	0.011 (2)	−0.006 (2)
C8	0.040 (3)	0.054 (3)	0.034 (3)	−0.011 (3)	0.006 (3)	−0.013 (2)
C9	0.030 (3)	0.058 (3)	0.034 (3)	0.002 (2)	0.003 (2)	0.003 (2)
C10	0.037 (3)	0.032 (3)	0.038 (3)	0.005 (2)	0.011 (2)	0.004 (2)
C11	0.040 (3)	0.089 (5)	0.066 (4)	−0.002 (3)	0.005 (3)	0.027 (4)
C12	0.077 (5)	0.051 (4)	0.134 (8)	0.032 (4)	0.014 (5)	0.002 (4)
N1	0.041 (2)	0.0216 (19)	0.032 (2)	0.0007 (16)	0.0073 (19)	0.0021 (15)
N2	0.048 (3)	0.0181 (16)	0.033 (2)	0.0054 (17)	0.004 (2)	−0.0015 (16)
N3	0.030 (2)	0.0252 (19)	0.029 (2)	0.0008 (15)	0.0041 (18)	0.0008 (15)
O1	0.0319 (18)	0.0387 (18)	0.0377 (19)	0.0067 (15)	0.0035 (15)	−0.0017 (15)
In1	0.03548 (19)	0.01872 (14)	0.03429 (18)	−0.00068 (16)	0.00671 (13)	−0.00053 (15)
Cl1	0.0634 (9)	0.0369 (6)	0.0565 (9)	0.0011 (6)	0.0239 (8)	0.0158 (6)
Cl2	0.0556 (9)	0.0504 (8)	0.0572 (9)	−0.0182 (7)	0.0048 (8)	−0.0175 (7)
Cl3	0.0470 (8)	0.0267 (6)	0.0591 (8)	0.0062 (5)	0.0170 (7)	−0.0020 (5)
S1	0.0354 (7)	0.0489 (8)	0.0454 (8)	0.0085 (6)	0.0092 (6)	0.0139 (6)

### Geometric parameters (Å, º)

**Table:**

C1—N1	1.362 (7)	C9—H9	0.9300
C1—C2	1.368 (8)	C10—N3	1.353 (6)
C1—H1	0.9300	C10—H10	0.9300
C2—C3	1.403 (7)	C11—S1	1.768 (6)
C2—H2	0.9300	C11—H11A	0.9600
C3—C4	1.350 (7)	C11—H11B	0.9600
C3—H3	0.9300	C11—H11C	0.9600
C4—C5	1.389 (7)	C12—S1	1.766 (7)
C4—H4	0.9300	C12—H12A	0.9600
C5—N1	1.347 (5)	C12—H12B	0.9600
C5—N2	1.390 (6)	C12—H12C	0.9600
C6—N3	1.342 (5)	N1—In1	2.279 (4)
C6—N2	1.380 (6)	N2—H2B	0.8600
C6—C7	1.401 (6)	N3—In1	2.267 (4)
C7—C8	1.367 (7)	O1—S1	1.531 (3)
C7—H7	0.9300	O1—In1	2.232 (3)
C8—C9	1.394 (8)	In1—Cl1	2.4381 (14)
C8—H8	0.9300	In1—Cl2	2.4535 (14)
C9—C10	1.361 (7)	In1—Cl3	2.4658 (13)
			
N1—C1—C2	122.8 (5)	H11B—C11—H11C	109.5
N1—C1—H1	118.6	S1—C12—H12A	109.5
C2—C1—H1	118.6	S1—C12—H12B	109.5
C1—C2—C3	118.4 (5)	H12A—C12—H12B	109.5
C1—C2—H2	120.8	S1—C12—H12C	109.5
C3—C2—H2	120.8	H12A—C12—H12C	109.5
C4—C3—C2	119.0 (5)	H12B—C12—H12C	109.5
C4—C3—H3	120.5	C5—N1—C1	117.9 (4)
C2—C3—H3	120.5	C5—N1—In1	125.2 (3)
C3—C4—C5	120.5 (4)	C1—N1—In1	116.2 (3)
C3—C4—H4	119.7	C6—N2—C5	129.8 (4)
C5—C4—H4	119.7	C6—N2—H2B	115.1
N1—C5—C4	121.2 (4)	C5—N2—H2B	115.1
N1—C5—N2	119.5 (4)	C6—N3—C10	118.0 (4)
C4—C5—N2	119.3 (4)	C6—N3—In1	125.2 (3)
N3—C6—N2	120.7 (4)	C10—N3—In1	116.7 (3)
N3—C6—C7	121.2 (4)	S1—O1—In1	121.04 (19)
N2—C6—C7	118.0 (4)	O1—In1—N3	83.32 (13)
C8—C7—C6	119.6 (4)	O1—In1—N1	85.12 (13)
C8—C7—H7	120.2	N3—In1—N1	79.63 (13)
C6—C7—H7	120.2	O1—In1—Cl1	89.31 (9)
C7—C8—C9	119.0 (5)	N3—In1—Cl1	93.18 (10)
C7—C8—H8	120.5	N1—In1—Cl1	171.37 (10)
C9—C8—H8	120.5	O1—In1—Cl2	89.15 (9)
C10—C9—C8	118.5 (5)	N3—In1—Cl2	168.87 (10)
C10—C9—H9	120.8	N1—In1—Cl2	91.60 (10)
C8—C9—H9	120.8	Cl1—In1—Cl2	94.92 (5)
N3—C10—C9	123.4 (4)	O1—In1—Cl3	171.97 (9)
N3—C10—H10	118.3	N3—In1—Cl3	90.74 (10)
C9—C10—H10	118.3	N1—In1—Cl3	88.50 (10)
S1—C11—H11A	109.5	Cl1—In1—Cl3	96.44 (5)
S1—C11—H11B	109.5	Cl2—In1—Cl3	95.91 (5)
H11A—C11—H11B	109.5	O1—S1—C12	103.1 (3)
S1—C11—H11C	109.5	O1—S1—C11	106.0 (3)
H11A—C11—H11C	109.5	C12—S1—C11	98.9 (4)
			
N1—C1—C2—C3	−0.7 (9)	C9—C10—N3—In1	172.6 (4)
C1—C2—C3—C4	−0.6 (8)	S1—O1—In1—N3	−128.7 (2)
C2—C3—C4—C5	−0.9 (8)	S1—O1—In1—N1	−48.6 (2)
C3—C4—C5—N1	3.8 (8)	S1—O1—In1—Cl1	138.0 (2)
C3—C4—C5—N2	−176.7 (5)	S1—O1—In1—Cl2	43.1 (2)
N3—C6—C7—C8	−4.3 (8)	C6—N3—In1—O1	53.4 (4)
N2—C6—C7—C8	175.6 (5)	C10—N3—In1—O1	−123.6 (3)
C6—C7—C8—C9	0.1 (8)	C6—N3—In1—N1	−32.9 (4)
C7—C8—C9—C10	1.7 (8)	C10—N3—In1—N1	150.2 (4)
C8—C9—C10—N3	0.5 (8)	C6—N3—In1—Cl1	142.3 (4)
C4—C5—N1—C1	−5.0 (7)	C10—N3—In1—Cl1	−34.6 (3)
N2—C5—N1—C1	175.5 (4)	C6—N3—In1—Cl2	5.6 (8)
C4—C5—N1—In1	165.3 (4)	C10—N3—In1—Cl2	−171.3 (4)
N2—C5—N1—In1	−14.3 (6)	C6—N3—In1—Cl3	−121.2 (4)
C2—C1—N1—C5	3.5 (8)	C10—N3—In1—Cl3	61.9 (3)
C2—C1—N1—In1	−167.6 (4)	C5—N1—In1—O1	−48.7 (4)
N3—C6—N2—C5	35.4 (8)	C1—N1—In1—O1	121.6 (4)
C7—C6—N2—C5	−144.5 (5)	C5—N1—In1—N3	35.3 (4)
N1—C5—N2—C6	−32.5 (7)	C1—N1—In1—N3	−154.3 (4)
C4—C5—N2—C6	147.9 (5)	C5—N1—In1—Cl2	−137.7 (4)
N2—C6—N3—C10	−173.5 (4)	C1—N1—In1—Cl2	32.6 (4)
C7—C6—N3—C10	6.4 (7)	C5—N1—In1—Cl3	126.4 (4)
N2—C6—N3—In1	9.6 (6)	C1—N1—In1—Cl3	−63.2 (4)
C7—C6—N3—In1	−170.5 (3)	In1—O1—S1—C12	153.0 (3)
C9—C10—N3—C6	−4.5 (7)	In1—O1—S1—C11	−103.7 (3)

### Hydrogen-bond geometry (Å, º)

**Table:**

*D*—H···*A*	*D*—H	H···*A*	*D*···*A*	*D*—H···*A*
C11—H11*C*···Cl2^i^	0.96	2.74	3.499 (8)	137

Symmetry code: (i) −*x*+1/2, *y*−1/2, −*z*+1/2.
